# Antifouling and pH-Responsive Poly(Carboxybetaine)-Based Nanoparticles for Tumor Cell Targeting

**DOI:** 10.3389/fchem.2019.00770

**Published:** 2019-11-22

**Authors:** Feng Ding, Shuang Yang, Zhiliang Gao, Jianman Guo, Peiyu Zhang, Xiaoyong Qiu, Qiang Li, Mingdong Dong, Jingcheng Hao, Qun Yu, Jiwei Cui

**Affiliations:** ^1^Key Laboratory of Colloid and Interface Chemistry of the Ministry of Education, School of Chemistry and Chemical Engineering, Shandong University, Jinan, China; ^2^State Key Laboratory of Microbial Technology, Shandong University, Qingdao, China

**Keywords:** poly(carboxybetaine methacrylate), PEG, pH-responsiveness, low-fouling, cell targeting

## Abstract

Nanocarriers with responsibility and surface functionality of targeting molecules have been widely used to improve therapeutic efficiency. Hence, we report the assembly of pH-responsive and targeted polymer nanoparticles (NPs) composed of poly(2-(diisopropylamino)ethyl methacrylate) (PDPA) as the core and poly(carboxybetaine methacrylate) (PCBMA) as the shell, functionalized with cyclic peptides containing Arginine-Glycine-Aspartic acid-*D*-Phenylalanine-Lysine (RGD). The resulting polymer NPs (PDPA@PCBMA-RGD NPs) can maintain the pH-responsivity of PDPA (pKa ~6.5) and low-fouling property of PCBMA that significantly resist non-specific interactions with RAW 264.7 and HeLa cells. Meanwhile, PDPA@PCBMA-RGD NPs could specifically target α_v_β_3_ integrin-expressed human glioblastoma (U87) cells. The pH-responsiveness and low-fouling properties of PDPA@PCBMA NPs are comparable to PDPA@poly(ethylene glycol) (PDPA@PEG) NPs, which indicates that PCBMA is an alternative to PEG for low-fouling coatings. The advantage of PDPA@PCBMA NPs lies in the presence of carboxyl groups on their surfaces for further modification (e.g., RGD functionalization for cell targeting). The reported polymer NPs represent a new carrier that have the potential for targeted therapeutic delivery.

## Introduction

Recently, low-fouling nanoparticles (NPs) with a specific targeting ability have attracted great attention in cancer therapy due to their improved therapeutic effects (Zhang et al., 2017; Ge et al., 2018; Trapiella-Alfonso et al., 2018; Wang et al., 2018). Considering the fact that targeting ligands and low-fouling coatings are typically modified on the surfaces of NPs and have competing functions (Ju et al., 2016), it is critical to balance the targeting and low-fouling properties to improve the therapeutic efficiency. To improve the low-fouling properties of NPs, poly(ethylene glycol) (PEG) is the most used polymer for surface modification (Yang et al., 2014, 2017; Wang J. et al., 2016) and could prevent the non-specific adsorption of proteins and phagocyte uptake (Cui et al., 2014, 2015).

Instead of PEG coatings, the Jiang group has pioneered the research using zwitterionic materials to avoid non-specific interactions (Ladd et al., 2008; Jiang and Cao, 2010; He et al., 2016) Zwitterionic materials with equimolarly mixed positive and negative charges [e.g., poly(sulfobetaine methacrylate) (PSBMA), poly(carboxybetaine methacrylate) (PCBMA), and poly(2-methacryloyloxyethyl phosphorylcholine) (PMPC)], have shown excellent low-fouling properties due to their strong electrostatic interactions with water molecules (Ladd et al., 2008; Yang et al., 2009; Carr et al., 2010; He et al., 2016; Zheng et al., 2017). Among these zwitterionic polymers, PCBMA showed an ultralow-fouling capacity, which could resist non-specific protein adsorption from undiluted blood plasma and serum and inhibit the formation of bacterial biofilms (Ladd et al., 2008; Cheng et al., 2009).

Zwitterionic PCBMA NPs could be engineered to increase blood circulation and the carboxyl groups in PCBMA be used for further functionalization (Zhang et al., 2011, 2012).

The stimuli-responsiveness of NPs is also desired for drug delivery, which could alter physiochemical properties (e.g., size, surface charge, and degradation) to improve the NPs accumulation at disease sites (Fu et al., 2018; Wu et al., 2018). Stimuli-responsive polymer NPs have shown their potential to encapsulate and protect therapeutic molecules during circulation and control the release of therapeutics in different triggers, such as pH, temperature, and redox species (Khaled et al., 2016; Yang et al., 2016; Gao et al., 2019). Therein, pH is one of the most commonly used triggers for controlled drug release (Zeng et al., 2017; Liu et al., 2018; Ma et al., 2019). These responsive NPs could be synthesized by interfacial polymerization or emulsion polymerization (Nagai et al., 2017; Truong et al., 2017; Zhang et al., 2019). Compared with conventional emulsion polymerization, surfactant-free emulsion polymerization is an efficient method to synthesize NPs in the absence of an added emulsifier. The combination of pH-responsive polymers and surfactant-free emulsion polymerization allows for the fabrication of functional polymer NPs for drug delivery (Zhao et al., 2013; Tian et al., 2015). For example, pH-responsive NPs composed of poly(2-(diethylamino)ethyl methacrylate)-co-p(2-aminoethyl methacrylate) PDEAEMA-co-PAEMA could deliver therapeutics into cells, and the pH-responsiveness property of these NPs could help the therapeutics escape from endosomes (Hu et al., 2007; Bayles et al., 2010). Poly(2-(diisopropylamino) ethyl methacrylate) (PDPA) is a pH-responsive polymer with a pKa of ~6.5, and it can be protonated to convert the polymer transition from hydrophobicity to hydrophilicity when the pH is below the pKa (Deng et al., 2005). Based on the pH-responsiveness of PDPA, various PDPA-based NPs have been designed for controlled intracellular degradation and drug release (Liang et al., 2012, 2014a,b; Xu et al., 2016; Zhou et al., 2016).

**Figure 1:**
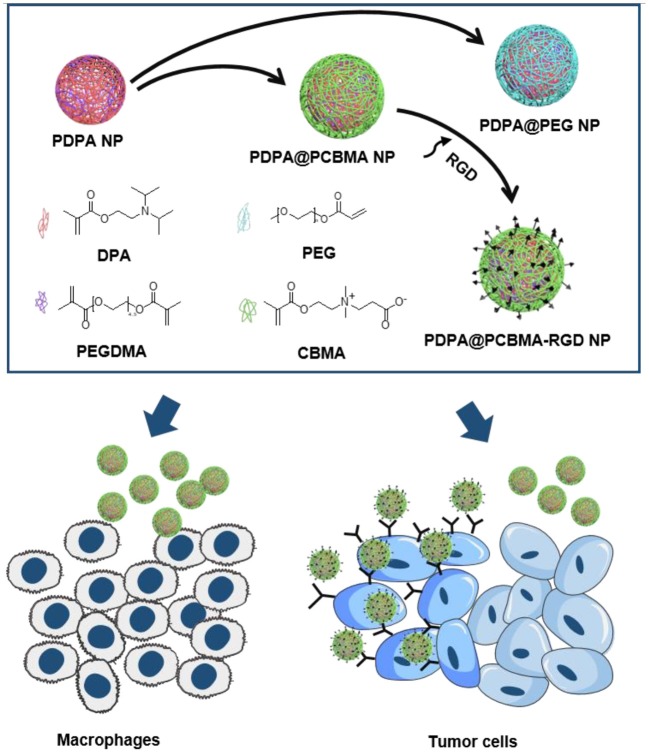
Illustration of the preparation of PDPA@PEG, PDPA@PCBMA, and PDPA@PCBMA-RGD NPs as well as the low-fouling property and targeting ability of PDPA@PCBMA-RGD NPs. Molecular structures of the monomers [DPA, 2-(diisopropylamino)ethyl methacrylate; PEG-acrylate, poly(ethylene glycol) methyl ether acrylate; CBMA, carboxybetaine methacrylate] and the cross-linker (PEGDMA, polyethylene glycol dimethacrylate).

In this study, pH-responsive and targeted polymer NPs with a core/shell structure were synthesized via surfactant-free emulsion polymerization for tumor cell targeting (Figure 1). The cores of polymer NPs were composed of PDPA, which was polymerized by the monomer of 2-(diisopropylamino) ethyl methacrylate (DPA) and cross-linked with poly(ethylene glycol) dimethacrylate (PEGDMA). The shell could be fabricated with either PCBMA or PEG to result in PDPA@PCBMA or PDPA@PEG NPs, respectively. Both NPs had similar pH-responsiveness and low-fouling properties against HeLa and RAW 264.7 cells. Targeting molecules of cyclic peptides containing Arginine-Glycine-Aspartic acid-*D*-Phenylalanine-Lysine (RGD) could be functionalized on PDPA@PCBMA NPs via the formation of amide bonds (Figure 1). The functionalized PDPA@PCBMA-RGD NPs could specifically target human glioblastoma (U87) cells expressed with α_v_β_3_ integrin. The reported targeted polymer NPs with pH-responsiveness have the potential for improved drug delivery.

## Materials and Methods

### Materials

2-(Diisopropylamino)ethyl methacrylate (DPA) (97%), Poly(ethylene glycol) methyl ether acrylate (PEG-acrylate) (M*n* 480), and ammonium peroxodisulfate (APS) were purchased from Sigma-Aldrich Chemical Co., Ltd. Polyethylene Glycol dimethacrylate (PEGDMA) and 2-(Dimethylamino)ethyl methacrylate (DMAEMA) was obtained from TCI (Shanghai) Development Co., Ltd. β-propiolactone was from J&K Scientific Ltd. RGD was purchased from GL Biochem (Shanghai) Ltd. 1,1′-Dioctadecyl-3,3,3′,3′-tetramethylindodicarbocyanine perchlorate (DID) was obtained from Tianjin Biolite Biotech Co., Ltd. Fetal bovine serum (FBS) was purchased from PAN Seratech. Dulbecco's modified eagle medium (DMEM) and Dulbecco's phosphate-buffered saline (DPBS) were obtained from Shijiazhuang Hongwei Biotechnology Co., Ltd. Thiazolyl blue tetrazolium bromide (MTT) was purchased from VWR Life Science. AF488-WGA and Hoechst 33342 were obtained from Thermo Fisher Scientific. All reagents were used as received without further purification.

### Characterization Methods

^1^H NMR spectra of the CBMA monomer were measured with a Bruker AVANCE400 instrument. The morphology of NPs was characterized by a JEOL JEM-1400 transmission electron microscope (TEM). The fluorescence of NPs was examined on a confocal laser scanning microscope (CLSM, LEICA TCS SP8). The fluorescence of cells was obtained using a fluorescent inverted microscope (OLYMPUS, IX 73). The hydrodynamic diameter and zeta-potential of NPs were measured with dynamic light scattering (DLS, Zetasizer Nano). The topography images of NPs were recorded in the air using an Asylum atomic force microscope (AFM, Cypher E5).

### Synthesis and Characterization of CBMA

A CBMA monomer was synthesized according to previously described methods (Figure S1) (Zhang et al., 2006). In brief, DMAEMA (5.0 g, 31.9 mmol) was dissolved in 200 mL of anhydrous acetone and then β-propiolactone (3.5 g, 48.3 mmol) in 20 mL of anhydrous acetone was added drop by drop. The reactant mixture was stirred under a nitrogen atmosphere at 4°C for 6 h. The precipitates were collected by filtration and washed with anhydrous acetone and anhydrous ether three times. The product was finally dried under vacuum at 30°C overnight. The isolated yield of the final product was a 56% (4.1 g) yield. ^1^H NMR (400 MH_Z_, D_2_O, ppm) (Figure S2): δ 6.06 (s, 1H), 5.68 (s,1H), 4.55 (t, 2H), 3.70 (t, 2H), 3.59 (t, 2H), 3.10 (s, 6H), 2.64 (t, 2H), and 1.84 (s, 3H).

### Preparation of PDPA@PCBMA NPs

PDPA NPs were synthesized according to the previously reported method with some alternations (Hu et al., 2007). Briefly, DPA (423 μL, 1.78 mmol), PEGDMA (3 μL, 0.01 mmol), and deionized water (15 mL) were added into a glass flask. The mixture was then emulsified with sonication for 5 min and equilibrated at 70°C for 25 min. Subsequently, the initiator of APS solution (150 μL, 200 mg/mL) was added into the reaction and kept at 70°C for 2 h to form the core of the PDPA NPs. To label PDPA NPs, DID (20 μL, 5 mg/mL) in anhydrous DMSO was added after the initiator was added. To fabricate PDPA@PEG NPs, PEG-acrylate (856.62 mg, 1.78 mmol) monomer was added into the PDPA NPs suspension synthesized above, and the suspension was stirred for 1.5 h to form a PEG corona. PDPA@PCBMA NPs were prepared similarly to the preparation of PDPA@PEG NPs, except that the CBMA monomer (409.18 mg, 1.78 mmol) were used as corona instead of PEG. To prepare targeted PDPA@PCBMA-RGD NPs, PDPA@PCBMA NPs (24 mg) were dispersed in 2 mL of PBS, then DMTMM (10 mg) was added with constant stirring for 30 min to active carboxyl groups before RGD (10 mg) was added to incubate for 24 h. The final products were purified by washing with PBS (pH 7.4) via centrifugation (50,000 *g*).

### pH-Responsiveness of Polymer NPs

The hydrodynamic diameter and zeta-potential of PDPA, PDPA@PEG, PDPA@PCBMA, and PDPA@PCBMA-RGD NPs at different pHs were monitored to investigate the pH-responsive properties. Briefly, samples were dispersed in 10 mM PBS of pH 4.0, 5.0, 5.5, 6.0, 6.5, 7.0, and 7.4, respectively. Then, the samples were kept overnight before the measurements for hydrodynamic diameter and zeta-potential were made.

### Cell Culture

HeLa, RAW 264.7, and U87 cells were cultured in DMEM with 10% (v/v) fetal bovine serum (FBS), 1% (v/v) penicillin and streptomycin. Cells were cultured with a complete medium at 37°C supplied with 5% CO_2_.

### Cell Association

HeLa or RAW 264.7 cells were seeded in a 24-well plate (5 × 10^4^ cells per well) and cultured overnight, allowing for cell attachment. Following this, DID-labeled PDPA, PDPA@PEG, and PDPA@PCBMA NPs at the concentration of 50 and 100 μg/mL were added and incubated for 4 or 10 h, respectively. After incubation, HeLa and RAW 264.7 cells were washed with DPBS and harvested by trypsin containing 0.25% EDTA. The cells were finally dispersed in DPBS with 2% FBS for flow cytometry experiments (ACEA, NovoCyte 3130).

### Cell Targeting of Polymer NPs

U87 cells were seeded in a 24-well plate with a density of 5 × 10^4^ cells per well in 500 μL DMEM medium and cultured at 37°C containing 5% CO_2_ humidified atmosphere overnight. DID-labeled PDPA@PCBMA and PDPA@PCBMA-RGD NPs were added and incubated for 4 h at 37°C. After incubation, cells were washed with DPBS and harvested by trypsin containing 0.25% EDTA. The cells were finally dispersed in 500 μL DPBS and analyzed using flow cytometry.

### Cell Imaging

U87 cells were seeded in a four-chambered confocal culture dish at a density of 5 × 10^4^ cells per well in 500 μL DMEM and cultured overnight. The original medium was then removed and fresh medium containing 15 μg/mL PDPA@PCBMA or PDPA@PCBMA-RGD NPs was added, and this was followed by 4 h incubation at 37°C. After incubation, cells were washed with DPBS and fixed with 4% paraformaldehyde for 10 min at room temperature, and this was followed by washing with DPBS. Cell nuclei were stained with Hoechst 33342 (1 μg/mL, 10 min incubation) and washed with DPBS three times. Cell membranes were stained with AF488-WGA (1 μg/mL) for 10 min and then washed three times with DPBS before imaging on a fluorescence microscope.

### Cell Viability Assay

*In vitro* cytotoxicity of PDPA, PDPA@PEG, and PDPA@PCBMA NPs was assessed using a 3-[4,5-dimethylthiazol-2-yl]-2,5-diphenyltetrazolium bromide (MTT) assay. HeLa and U87 cells were seeded in 96-well plates (1 × 10^4^ cells per well) and cultured overnight for adhere. Fresh media containing different concentrations of polymer NPs were added and cultured at 37°C in a 5% CO_2_ humidified atmosphere. After being incubated for 24 or 48 h, fresh DMEM (100 μL) and 10 μL MTT (5 mg/mL in DPBS) was added and incubated for an additional 4 h. The medium was removed and 100 μL of dimethyl sulfoxide (DMSO) was added to each well to dissolve the formazan crystals. The absorbance at 570 nm was measured by a plate reader (TECAN, SPARK 10 M).

## Results and Discussions

### Preparation and Characterization of Polymer NPs

**Figure 2:**
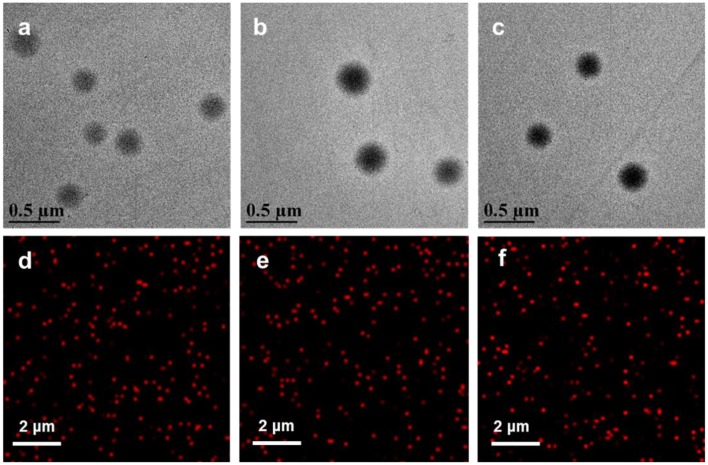
TEM and CLSM images of **(a,d)** PDPA, **(b,e)** PDPA@PEG, and **(c,f)** PDPA@PCBMA. Red fluorescence was from the encapsulated DID dyes.

For the synthesis of PDPA@PEG NPs, the cores of PDPA NPs were fabricated using DPA for monomers and PEGDMA for cross-linkers via surfactant-free emulsion polymerization. A PEG-acrylate monomer was added into the reaction system to form a PEG corona on PDPA NPs (PDPA@PEG NPs). For the synthesis of PDPA@PCBMA NPs, a CBMA monomer was used with the same procedure. The cores of PDPA NPs with lots of tertiary amine could protonate to change the surface charge in acidic environments and the shell composed of PEG or PCBMA could improve the low-fouling property. The size distribution and zeta-potential of the polymer NPs were monitored by a Zetasizer. As shown in Table 1, the average hydrodynamic diameter of PDPA NPs was about 170 nm. After polymerization with PCBMA and PEG, the size changed to be 228 and 236 nm, respectively. Most importantly, all of these NPs had a narrow size distribution (PDI 0.03–0.07). PDPA NPs dispersed in PBS buffer (10 mM, pH 7.4) were neutrally charged while PDPA@PEG (−4 mV) and PDPA@PCBMA (−8 mV) NPs were slightly negatively charged, and this could be indicative of low cell internalization compared with positively charged nanocarriers (Sun et al., 2016; Wang S. et al., 2016). When the RGD was modified on the surface of the PDPA@PCBMA NPs, the size was 330 nm and ζ-potential was nearly neutral as well (−1.3 mV). As shown in TEM images (Figures 2a–c), these polymer NPs were monodisperse. The average diameter of PDPA NPs observed in the TEM was about 190 nm, while the average diameter of the PDPA@PEG and PDPA@PCBMA NPs was about 240 nm. The diameter was slightly large compared to the DLS results, which could be caused by NP collapse during air drying for TEM observation. The NP collapse was proved by an AFM, where height was <40 nm, much smaller than the diameter of the polymer NPs (Figure S3). CLSM images (Figures 2d–f) further indicated that these polymer NP-encapsulated DID dyes were monodisperse and well-dispersed in the PBS buffer, where DID was used as a hydrophobic model drug.

**Table 1:** Characterization of PDPA, PDPA@PEG, PDPA@PCBMA, and PDPA@PCBMA-RGD NPs.

	**Size (nm)**	**Zeta-potential (mV)**	**PDI**
PDPA	175.1 ± 2	−1.6 ± 0.3	0.03 ± 0.01
PDPA@PEG	228.6 ± 5	−4.2 ± 0.3	0.07 ± 0.03
PDPA@PCBMA	236.8 ± 4	−8.2 ± 0.1	0.05 ± 0.01
PDPA@PCBMA-RGD	334.5 ± 3	−1.28 ± 0.1	0.17 ± 0.01

### The pH-Responsiveness of the Polymer NPs

**Figure 3:**
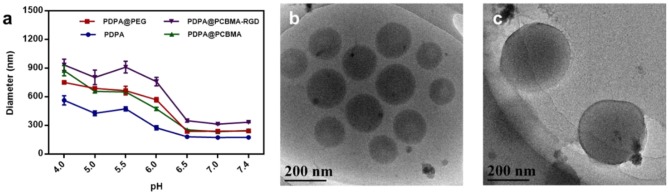
**(a)** Diameter changes of PDPA, PDPA@PEG, PDPA@PCBMA, and PDPA@PCBMA-RGD NPs cultured in 10 mM PBS buffer at a different pH. Cryo-TEM images of PDPA@PCBMA NPs at **(b)** pH 7.4 and **(c)** 5.0 in a 10 mM PBS buffer.

To investigate the pH-responsiveness of polymer NPs, the hydrodynamic diameters and zeta-potential of these polymer NPs were monitored after incubation in a buffer with a different pH. As shown in Figure 3a, the diameter of the polymer NPs increased with the decrease of pH, which was due to the protonation of a tertiary amine group of PDPA when the pH value was lower than its pKa (~6.4). Intermolecular repulsion due to the positively charged PDPA could induce the stretching of NPs and diameter increase (Xu et al., 2016; Zhou et al., 2016). The diameter of the PDPA NPs at pH 4.0 was three-fold higher compared to that at pH 7.4. PDPA@PEG, PDPA@PCBMA, and PDPA@PCBMA-RGD NPs swelled about four times at pH 4.0 compared to at pH 7.4. Cryo-TEM was also used to investigate the pH-responsiveness of polymer NPs. Figures 3b,c show that the size of the PDPA@PCBMA NPs increased from 190 to 320 nm as the pH value decreased from 7.4 to 5.0. However, the size of PDPA@PCBMA NPs obtained by cryo-TEM is smaller than the results from DLS, which was due to the formation of a hydration layer in the aqueous medium (He et al., 2016). Similarly, the zeta potential of polymer NPs reversed from a negative charge to a positive charge as the pH value decreased from 7.4 to 4.0 (Figure S4). The charge reversal property of these NPs was mainly caused by the protonation of a tertiary amine group in PDPA polymers. This reversed charge could improve cell association when these NPs are in a tumor microenvironment (Sun et al., 2016).

### Cell Association

**Figure 4:**
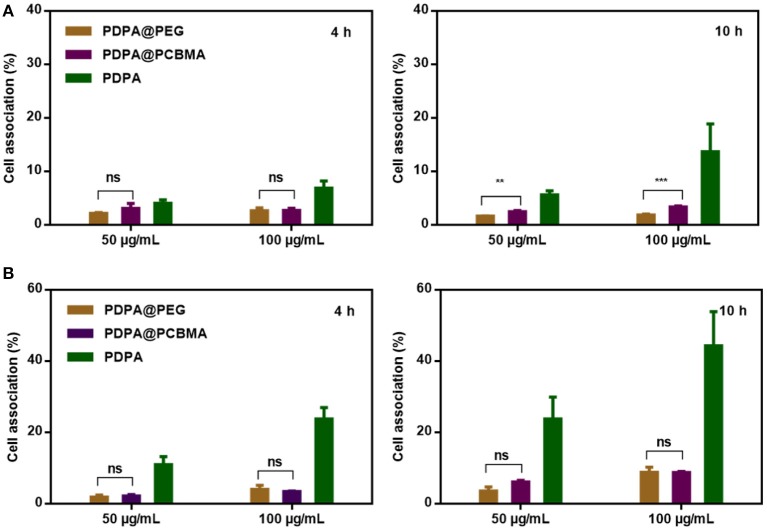
Association of PDPA, PDPA@PEG, and PDPA@PCBMA NPs with **(A)** HeLa and **(B)** RAW 264.7 cells after 4 and 10 h incubation at 37°C, respectively (**p < 0.01, *** *p* < 0.001).

HeLa and RAW 264.7 cells were used to investigate the low-fouling property of polymer NPs. The cytotoxicity of PDPA, PDPA@PEG, and PDPA@PCBMA NPs was found to be negligible at a concentration of 100 μg/mL, according to MTT assays (Figure S5). Compared with HeLa cells, RAW 264.7 cells are macrophages and have a stronger phagocytosis of NPs. As shown in Figure 4A, <3% of PDPA@PEG and PDPA@PCBMA NPs were associated with HeLa cells after 4 and 10 h of incubation; even the concentration of the NPs was as high as 100 μg/mL. However, more than 15% of PDPA NPs were associated with HeLa cells after 10 h of incubation. When the NPs were incubated with RAW 264.7 cells, the cell association of PDPA@PEG and PDPA@PCBMA NPs was significantly lower than of PDPA NPs (Figure 4B). For example, the cell association of PDPA@PEG and PDPA@PCBMA NPs was <10% when the incubation time was 10 h and NP concentration was 100 μg/mL. However, more than 45% of PDPA NPs were associated with RAW 264.7 cells. These results showed the low-fouling property of PDPA@PCBMA NPs were comparable with PDPA@PEG NPs. However, compared with PDPA@PEG NPs, PDPA@PCBMA NPs containing lots of carboxyl groups are easily modified with functional molecules.

### Cell Targeting of PDPA@PCBMA-RGD NPs

**Figure 5:**
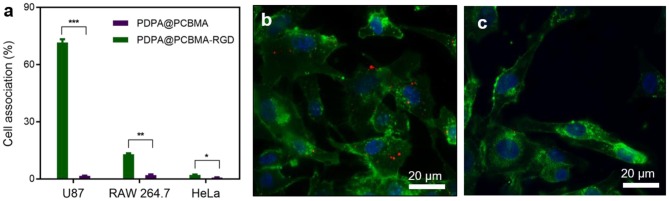
**(a)** Cell targeting of PDPA@PCBMA-RGD NPs to U87 cells (*p < 0.05, **p < 0.01, ***p < 0.001). Fluorescence microscopy images of the cell interaction with **(b)** PDPA@PCBMA-RGD NPs and **(c)** PDPA@PCBMA NPs. Cell membranes and nuclei were stained with AF488–WGA (green) and Hoechst 33342 (blue), respectively. NPs were labeled with the encapsulated DID (red).

Low-fouling NPs could resist non-specific interactions with macrophages but also diseased cells. Therefore, it is critical to functionalize the low-fouling NPs with targeting ligands to improve specific cell targeting. RGD peptides have specific interactions with α_v_β_3_ integrins, which are overexpressed in many cancer cells, such as HepG2 and U87 cells (Huang et al., 2014; Belhadj et al., 2017; Cui et al., 2018). Herein, RGD was modified on PDPA@PCBMA NPs via the formation of amide bonds. To investigate the targeting capacity, the cell association of PDPA@PCBMA NPs with U87 cells was investigated, and HeLa cells that do not express α_v_β_3_ integrins were used as controls. U87 and HeLa cells were cultured with PDPA@PCBMA and PDPA@PCBMA-RGD NPs at a concentration of 15 μg/mL for 4 h. As shown in Figure 5a, PDPA@PCBMA-RGD NPs had a high association (~70%) with U87 cells, while the NPs had <15 and 3% association with RAW 264.7 and HeLa cells, respectively. The results suggest that PDPA@PCBMA-RGD NPs could specifically target U87 cells while maintaining the low-fouling property against HeLa cells. Fluorescence microscopy images also confirmed the results of the flow cytometry results that PDPA@PCBMA-RGD NPs (red) could target U87 cells instead of HeLa cells (Figures 5b,c, Figure S6). These results indicate that PDPA@PCBMA-RGD NPs could effectively target α_v_β_3_ integrin-overexpressed U87 cells, which shows their potential for targeted drug delivery.

## Conclusions

In summary, we reported the assembly of PDPA@PCBMA-RGD NPs via surfactant-free emulsion polymerization using PDPA as the core and PCBMA as the shell for tumor cell targeting. In addition, hydrophobic model drugs (i.e., DID molecules) can be encapsulated during NP preparation. The core of the PDPA could be protonated or deprotonated when the environmental pH was below or above its pKa (~pH 6.5), respectively. The shell of the PCBMA is used to endow PDPA@PCBMA NPs with low-fouling property, which is similar to PDPA@PEG NPs. Both PDPA@PCBMA and PDPA@PEG NPs can maintain the pH-responsivity of the PDPA and low-fouling property of either PCBMA or PEG, which have low interactions with HeLa and RAW 264.7 cells. Different with PDPA@PEG NPs, PDPA@PCBMA NPs can be further functionalized with a targeting molecule of RGD. The obtained PDPA@PCBMA-RGD NPs could resist non-specific interactions with HeLa cells with a low expression of α_v_β_3_ integrins but could specifically target U87 cells with high expression of α_v_β_3_ integrins. This work demonstrates the potential of zwitterionic PCBMA as a good substitute to PEG due to its excellent low-fouling property and the carboxyl groups for further functionalization. The reported PDPA@PCBMA-RGD NPs with low-fouling and targeting properties are good candidates to investigate bio-nano interactions and could potentially be used for therapeutic delivery.

## Data Availability Statement

All datasets generated for this study are included in the article/Supplementary Material.

## Author Contributions

FD, JH, QY, and JC conceived the ideas. FD, ZG, and JG performed cell experiments. SY, PZ and XQ synthesized and characterized CBMA. QL and MD conducted the AFM experiments. FD, QY, and JC drafted the manuscript. All authors discussed the results and commented on the manuscript.

### Conflict of Interest

The authors declare that the research was conducted in the absence of any commercial or financial relationships that could be construed as a potential conflict of interest.
