# Supplementary material for: Antifouling and pH-Responsive Poly(Carboxybetaine)-Based Nanoparticles for Tumor Cell Targeting
Source: Front Chem. 2019 Nov 22;7:770. doi: 10.3389/fchem.2019.00770 (PMC6883901; doi:10.3389/fchem.2019.00770)
Supplement: Supplementary file 1 [file Data_Sheet_1.pdf]

Antifouling and pH-responsive poly(carboxybetaine)-based nanoparticles for tumor cell targeting

Feng Ding[1], Shuang Yang[1], Zhiliang Gao[1], Jianman Guo[1], Peiyu Zhang[1], Xiaoyong Qiu[1], Qiang Li[1], Mingdong Dong[1], Jingcheng Hao, Qun Yu[1,*], Jiwei Cui[1,2,*]

[1]Key laboratory of Colloid and Interface Chemistry of the Ministry of Education, School of Chemistry and Chemical Engineering, Shandong University, Jinan, Shandong 250100, China.

[2]State Key Laboratory of Microbial Technology, Shandong University, Qingdao, Shandong 266237, China

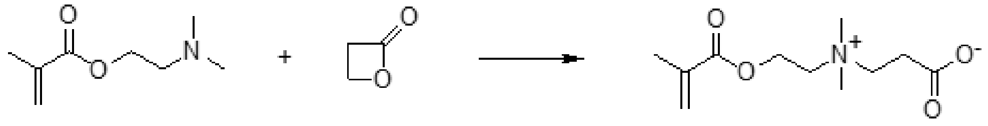

Figure S1. Synthesis of CBMA.

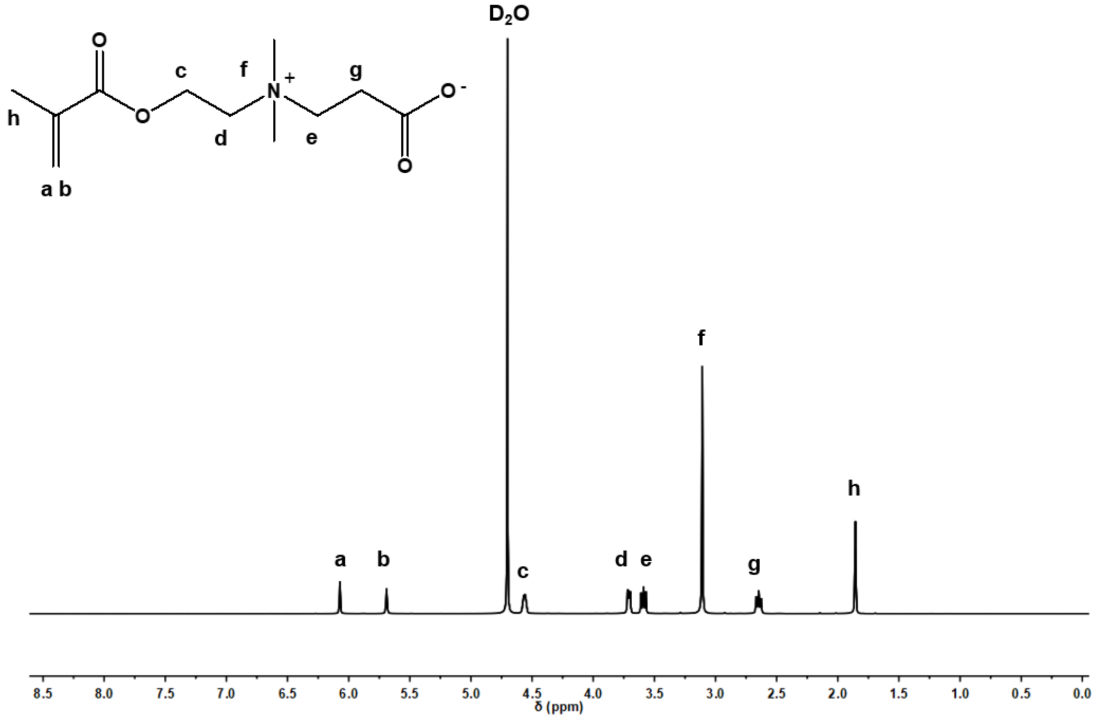

Figure S2. ^{1}H NMR spectrum of CBMA in D_2O.

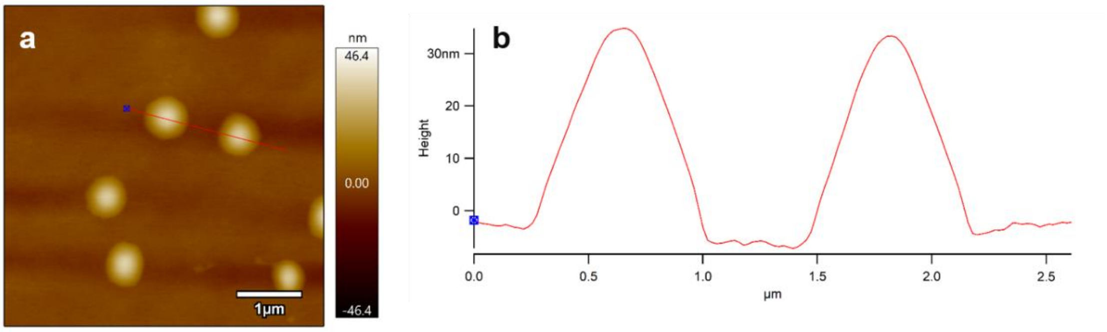

Figure S3. (a) AFM image of PDPA@PCBMA NPs. (b) Height profiles of NPs, marked with red line in Figure S3a.

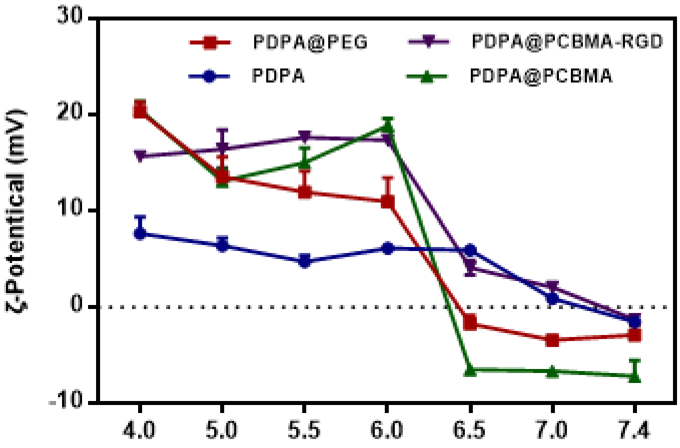

Figure S4. Zeta-potential of PDPA, PDPA@PEG, PDPA@PCBMA and PDPA@PCBMA-RGD NPs incubated with 10 mM PBS buffer at different pH.

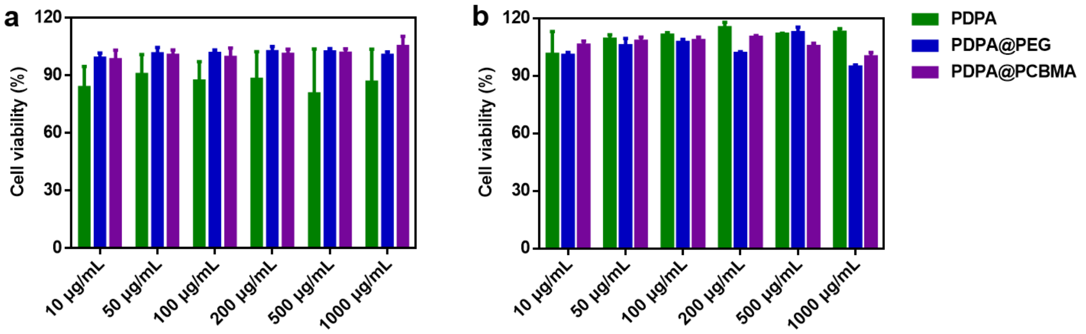

Figure S5. Cell viability of HeLa cells after incubation with PDPA, PDPA@PEG and PDPA@PCBMA NPs for (a) 24 and (b) 48 h, respectively.

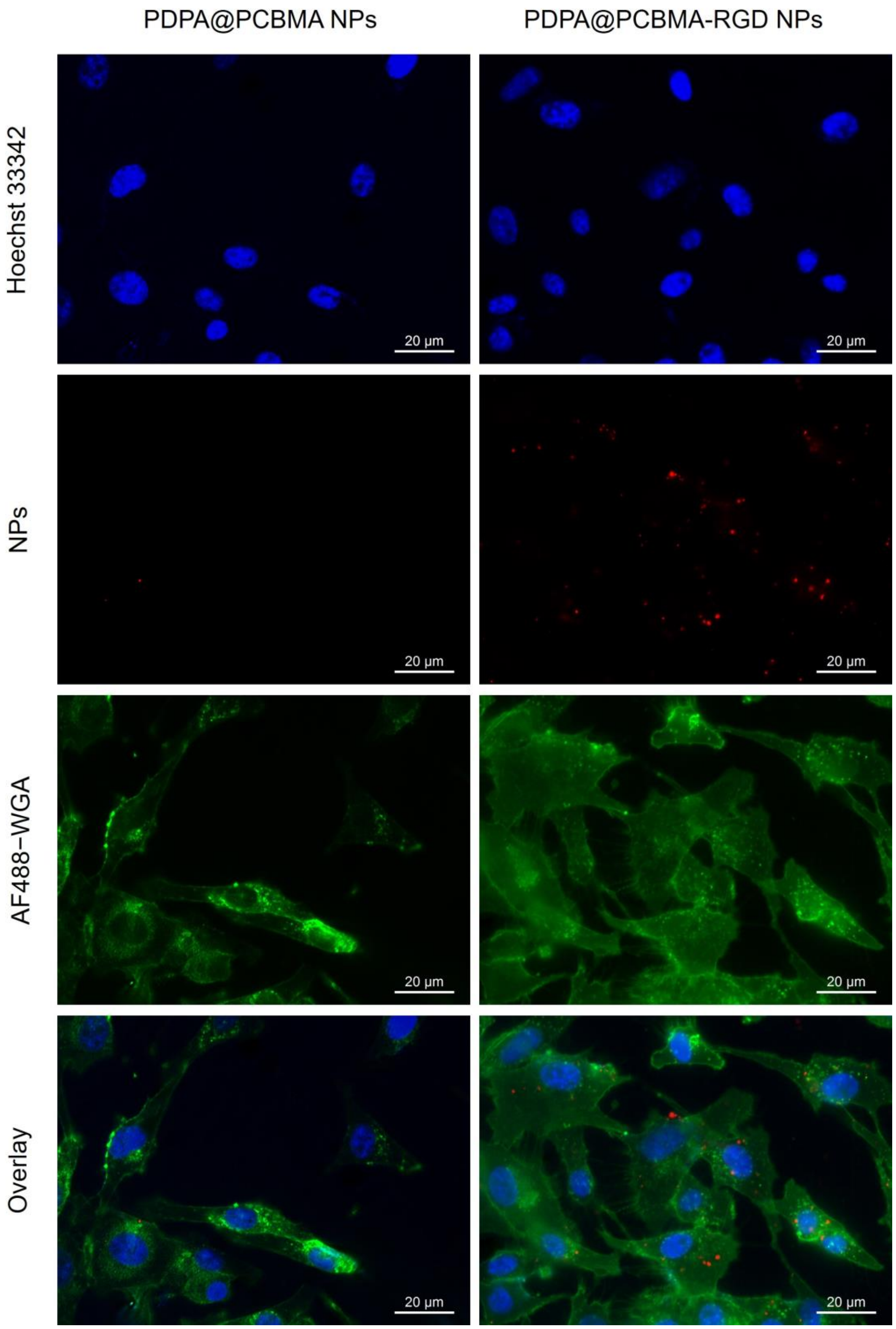

Figure S6. Fluorescence microscopy images of the cell interaction with PDPA@PCBMA and PDPA@PCBMA-RGD NPs. Cell members and nuclei were stained with AF488−WGA (green), and Hoechst 33342 (blue), respectively. NPs were labeled with the encapsulated DID (red).
